# Type‐specific clinical characteristics of adenovirus‐associated influenza‐like illness at five US military medical centers, 2009–2014

**DOI:** 10.1111/irv.12392

**Published:** 2016-05-13

**Authors:** Michael A. Koren, John C. Arnold, Mary P. Fairchok, Tahaniyat Lalani, Patrick J. Danaher, Christina M. Schofield, Michael Rajnik, Erin A. Hansen, Deepika Mor, Wei‐Ju Chen, Michelande Ridoré, Timothy H. Burgess, Eugene V. Millar

**Affiliations:** ^1^ Walter Reed National Military Medical CenterWashingtonDCUSA; ^2^ Naval Medical CenterSan DiegoCAUSA; ^3^ Infectious Disease Clinical Research ProgramDepartment of Preventive Medicine and BiostatisticsUniformed Services University of the Health SciencesBethesdaMDUSA; ^4^ Henry M. Jackson Foundation for the Advancement of Military MedicineRockvilleMDUSA; ^5^ Madigan Army Medical CenterFort LewisWAUSA; ^6^ Naval Medical CenterPortsmouthVAUSA; ^7^ San Antonio Military Health SystemSan AntonioTXUSA; ^8^ Naval Health Research CenterSan DiegoCAUSA; ^9^ Children's National Medical CenterWashingtonDCUSA

**Keywords:** Adenovirus, influenza‐like illness, military

## Abstract

**Background:**

Adenovirus is a recognized cause of influenza‐like illness (ILI). The proportion of ILI attributable to adenovirus is not known. Moreover, knowledge gaps remain with respect to the epidemiologic, virologic, and clinical characteristics of adenovirus‐associated ILI among otherwise healthy individuals.

**Methods:**

An observational, longitudinal study of <65‐year‐old patients with febrile ILI at five medical centers was conducted from 2009 to 2014. Nasopharyngeal specimens obtained at enrollment were first tested by single‐reaction PCR for adenovirus, then further evaluated by a multiplex PCR assay for other respiratory viral pathogens. Symptoms over a 28‐day period were collected.

**Results:**

We enrolled 1536 individuals, among whom 43 (2·8%) were positive for adenovirus. The median age of cases was 3·4 years (range: 4 months to 41 years). Three were hospitalized. Species and serotype information was available for 33 (76·7%) cases. Species C (*n* = 21) was the most common, followed by B3 (*n* = 9) and one each of E4a, D46, and A. Species C infections were more frequent in children (*P* < 0·01). Half of the cases were positive for at least one other respiratory viral pathogen. Symptoms were generally mild and most commonly included cough (90%), fatigue (79%), rhinorrhea (74%), loss of appetite (71%), and sore throat (64%). Children with non‐C adenovirus infection were more likely to report sore throat (*P* = 0·05) and hoarseness (*P* = 0·06) than those with species C infection.

**Conclusions:**

Adenovirus is frequently detected with other respiratory viruses. Persons with non‐C adenovirus infections reported more severe symptoms, suggesting there may be species‐specific differences in virulence and/or host response to infection.

## Introduction

Adenoviruses are a recognized cause of acute respiratory infection. They have considerable antigenic diversity, with >54 unique serotypes having been described.^1^ The severity of adenovirus‐associated respiratory infections varies considerably: Adenovirus has been isolated from asymptomatic individuals^2^ or largely associated with mild upper respiratory illness.^3^ By contrast, adenovirus has been implicated in outbreaks of severe respiratory disease among military trainees, some with fatal outcomes.^4^, ^5^ The heightened risk of adenovirus infection among military recruits stimulated the initial development, as well as the reintroduction, of an adenovirus vaccine specifically for these populations. Routine use of adenovirus vaccine at military training centers has led to significant declines in overall rates, as well as dramatic shifts in the etiologic burden, of febrile respiratory illness.^6^, ^7^

Large‐scale, population‐based studies have described the various clinical manifestations of adenovirus infection and serotype‐specific differences in severity at the time of clinical presentation.^8^, ^9^ More recent studies of adenovirus‐associated respiratory infections have been done; however, these were restricted to hospitalized children.^10^ To our knowledge, few published studies of adenovirus‐associated respiratory infection have utilized multiplex diagnostic assays, suggesting that while adenovirus may have been associated with illness, the influence of a coinfecting viral pathogen on symptom severity could not be ruled out. Herein we describe the burden and clinical characteristics of adenovirus‐associated influenza‐like illness among otherwise healthy children and adults at United States military hospitals from 2009 to 2014.

## Methods

### Study setting and participant eligibility

The Acute Respiratory Infection Consortium (ARIC) is a multicenter, multidisciplinary clinical research network for the study of influenza‐like Illness (ILI) among otherwise healthy United States military personnel and beneficiaries. The ARIC natural history study was created to determine the etiology, epidemiology, and clinical characteristics of ILI among those presenting for care at five US‐based military treatment facilities. We recruited patients <65 years presenting within 72 hour of onset of fever (temperature ≥100·4°F) in addition to one of the following symptoms: cough, sputum production, shortness of breath, chest pain, and/or sore throat.^11^ Symptom severity—scored as none (0), mild (1), moderate (2), or severe (3)—was ascertained by in‐person interviews conducted with study participants on the day of enrollment (day 0) and during follow‐up visits on study days 3, 7, and 28. Symptom severity among participants <4 years was assessed by observation rather than self‐report.

Written informed consent was obtained at enrollment. The study was approved by the Infectious Disease Institutional Review Board of the Uniformed Services University of the Health Sciences (IDCRP‐045).

### Specimen collection and molecular analysis

Nasopharyngeal swabs (FLOQSwabs^™^, Copan Diagnostics, Corona, CA) were obtained on the day of enrollment and processed by adenovirus‐specific PCR via two methods. Samples received prior to October 11, 2010 were extracted using the QIAamp 96 DNA Blood Kit (Qiagen, Venlo, the Netherlands). Samples received after that date were extracted on the MagNA Pure LC 2·0 instrument using the Total Nucleic Acid kit (Roche Diagnostics, Basel, Switzerland). Adenovirus detection and typing was done using the Applied Biosystems 7500 Fast Real‐Time PCR system (Life Technologies, Carlsbad, CA) using a laboratory‐developed adenovirus real‐time PCR assay that targets the penton region. This assay has been validated against a previously described, CAP‐accredited PCR assay (Appendix 1).^12^ Adenovirus‐positive samples were subsequently tested by a multiplex diagnostic panel (Diatherix TEM‐PCR; Diatherix Laboratories, Inc.; Huntsville, AL) that included primers for common viral respiratory pathogens, including adenovirus, influenza, parainfluenza, rhinovirus, coronavirus, respiratory syncytial virus, human metapneumovirus, and Coxsackie virus.

### Data analysis

Distributions of participant demographics, geographic location, and potential risk factors (all categorical variables) were compared by adenovirus type (type C and non‐C) using the Pearson chi‐square test or Fisher's exact test where appropriate.. Symptoms were analyzed and median symptoms scores were calculated and compared by adenovirus type using Wilcoxon rank‐sum test. Composite symptom scores in the following four categories were calculated as the sum of individual symptom score in each category: (i) upper respiratory score: earache, runny nose, sore throat, and sneezing; (ii) lower respiratory score: cough, breathing difficulty, hoarseness, and chest pain; (iii) systemic score: muscle ache, fatigue, headache, and chills; and (iv) total score: sum of the above three categories. Median composite scores were compared between cases with type C and non‐type C using Wilcoxon rank‐sum test. A review of symptoms over time was also undertaken by evaluating patient symptoms at each visit. Overall median symptom scores for adenovirus infections were calculated and compiled for each symptom for each respective visit. *P*‐values less than 0·05 were considered statistically significant.

Analyses were performed using SAS software, version 9·3 (SAS Institute, Cary, North Carolina).

## Results

**Figure 1:**
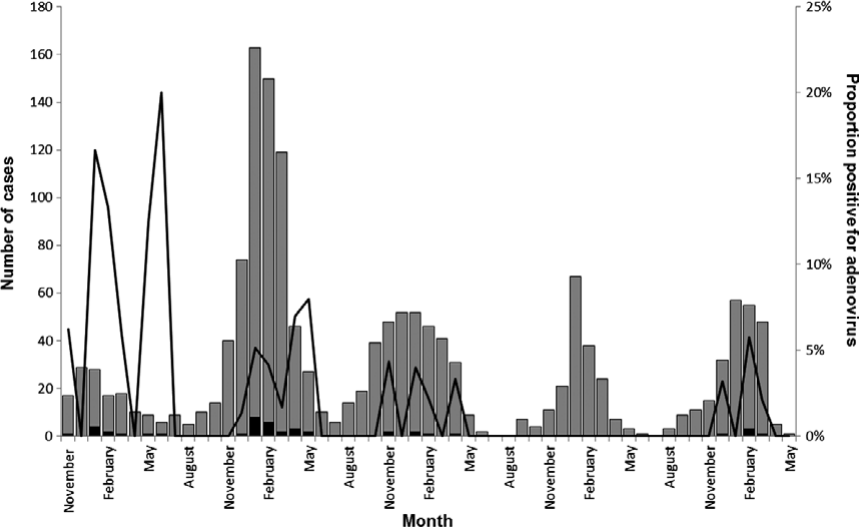
Distribution of adenovirus‐positive cases among patients with influenza‐like illness, by month and year. Gray bars represent the total number of cases of influenza‐like illness. Black bars represent the number of adenovirus‐positive cases. Black indicates the proportion of cases positive for adenovirus.

Of 1537 patients enrolled from September 2009–May 2014, 43 (2·8%) were positive for adenovirus. Cases occurred from November to June, with the highest (32·6%) number of cases occurring in the month of January (Figure 1). The majority (75%) of cases were <18 years; 60% were <5 years (Table 1). Fifty‐one percent were male. Three cases were enrolled as inpatients. There were no deaths. Baseline symptoms were generally mild and most commonly included cough (90%), fatigue (79%), rhinorrhea (74%), loss of appetite (71%), and sore throat (64%).

**Table 1:** Demographic characteristics of patients with adenovirus‐associated influenza‐like illness, by species

Characteristics	Adenovirus Species			*P* a
	Type C *n* = 21	Non‐C *n* = 12	Unknown *n* = 10	
Age (years)				
<18	20 (95·2)	6 (50·0)	5 (55·6)	<0·01
≥18	1 (4·8)	6 (50·0)	4 (44·4)	
Sex				
Male	10 (47·6)	6 (50)	5 (55·6)	0·90
Female	11 (52·3)	6 (50)	4 (44·4)	
Study site				
SAMHS, San Antonio, TX	7 (33·3)	2 (16·6)	0 (0)	0·65
NMCSD, San Diego, CA	8 (38·1)	7 (58·3)	4 (44·4)	
NMCP, Portsmouth, VA	3 (14·3)	2 (16·6)	5 (55·6)	
MAMC, Tacoma, WA	3 (14·3)	1 (8·3)	0 (0)	
Race				
Caucasian	16 (76·2)	11 (91·6)	9 (100)	0·65
African American	3 (14·3)	1 (8·3)	0 (0)	
Other	2 (9·5)	0 (0·0)	0 (0)	
Military status				
Active duty	1 (4·8)	6 (50·0)	6 (66·7)	<0·01
Family members	20 (95·2)	6 (50·0)	3 (33·3)	
Smoker in the household				
Yes	2 (9·5)	4 (33·3)	5 (55·6)	0·16
No	19 (90·5)	8 (66·6)	4 (44·4)	
Daycare exposure				
Yes	9 (43·9)	6 (50·0)	4	0·73
No	12 (57·1)	6 (50·0)	5	
Hospitalization				
Yes	0 (0·0)	2 (16·6)	1	0·13
No	21 (100·0)	10 (8·3)	8	

a Chi‐square test comparing characteristics of type C versus non‐type C infections.

Adenovirus species and serotype information was available for 34 (79·1%) cases. Adenovirus species C was most common (*n* = 21; 63·6%), followed by B/3 (*n* = 9; 27·3%), and one each of A, D/46, and E/4a. One patient was positive for C and E/4a. Species C was more frequent among participants <18 years (Table 1). There were no other significant differences in participant characteristics with respect to the distribution of C and non‐C adenovirus species.

Among all cases, 22 (51·2%) were positive for another respiratory virus, including influenza (*n* = 7; 31·8%), respiratory syncytial virus (*n* = 6; 27·3%), rhinovirus (*n* = 6; 27·3%), and coronavirus (*n* = 5; 22·7%). The proportion of cases with a codetected respiratory virus was highest among children 5–17 years (66·7%) and children <5 years (57·7%), as compared to 27·3% of adults (Table 2). The prevalence of viral codetection did not significantly differ between individuals with species B versus species C adenovirus infection (33·3% versus 66·7%, respectively; *P* = 0·123).

**Table 2:** Number and type of codetected viral respiratory pathogens among cases with adenovirus‐associated influenza‐like illness, by age‐group

Codetected viruses	Age‐group, years			Total (*n* = 43)
	<5 (*n* = 26)	5–17 (*n* = 6)	≥18 (*n* = 11)	
Influenza	4	3	0	7
RSV	3	1	1	5
Parainfluenza	0	0	1	1
Rhinovirus	4	0	0	4
Coronavirus	2	0	1	3
Corona/HRV/RSV	1	0	0	1
Coronavirus/HRV	1	0	0	1
Number (%) of codetections	15 (57·7)	4 (66·7)	3 (27·3)	22 (51·1)

When serotype C and non‐C adenovirus infections were compared, several associations were noted (Table 3). First, adenovirus C was more common among children (*P* < 0·01). Non‐type C‐infected patients had more severe sore throat compared to type C (median score of 3 versus 0; Table 4). The frequency and severity of headache, joint pain, and muscle aches was higher among those with non‐C adenovirus infections. Moreover, the composite systemic symptom score was higher among those with non‐C adenovirus infection (*P* = 0·03).

**Table 3:** Comparison of symptom severity at enrollment by adenovirus type

	Species C *N* = 21	Non‐species C *N* = 12	*P*‐value		
	Median, *N*	Median, *N*	All	Children	Adults
Upper respiratory symptoms					
Sneezing	1·00 (21)	0·0 (12)	0·72	0·35	0·25
Sore throat	0·00 (10)	3·0 (9)	0·003	0·07	0·79
Earache	0·0 (10)	0·0 (9)	0·39	0·13	0·25
Rhinorrhea	2·00 (21)	1·0 (12)	0·77	0·98	1·00
Cough	1·00 (21)	1·0 (12)	0·55	0·70	0·80
Hoarseness	0·00 (21)	1·0 (12)	0·03	0·10	0·46
Lower respiratory symptoms					
Shortness of breath	0·00 (19)	0·0 (12)	0·30	0·10	0·77
Chest pain	0·0 (10)	0·0 (9)	0·04	1·00	0·60
Gastroenteritis symptoms					
Decreased appetite	2·00 (21)	1·0 (12)	0·73	0·85	0·62
Vomiting	0·00 (21)	0·0 (12)	0·91	0·86	1·00
Diarrhea	0·00 (21)	0·0 (12)	0·59	0·46	0·76
Abdominal pain	0·00 (11)	0·0 (9)	0·57	0·53	0·58
Systemic symptoms					
Headache	0·00 (10)	1·0 (9)	0·009	0·14	0·58
Red eyes	0·00 (21)	0·0 (12)	0·41	0·96	0·58
Chills	0·00 (20)	1·0 (12)	0·09	0·79	0·46
Nausea	0·00 (10)	0·0 (9)	0·92	0·51	0·60
Joint pain	0·00 (7)	1·0 (9)	0·02	0·27	NA
Muscle aches	0 (10)	1·5 (9)	0·008	0·13	0·31
Fatigue	2·00 (21)	2·0 (12)	0·13	0·53	1·00
Dizziness	0·00 (10)	0·50 (9)	0·44	0·81	0·80
Composite severity score (ARIC original), Med (IQR)					
Respiratory symptom score	1·0 (1·5–1·0)	1·3 (0·85–1·6)	0·12	0·76	0·61
Systemic symptom score	1·0 (0·2–1·0)	1·2 (0·9–2·2)	0·03	0·44	0·30
Modified Hayden severity score, Med (IQR)^a^					
Upper respiratory	2 (0–5)	4 (3–4)	0·40	0·37	0·26
Lower respiratory	1 (1–2)	3 (1–4)	0·40	1·00	0·61
Systemic	4 (0–11)	6 (6–10)	0·64	0·77	0·29
Total	7 (2–17)	14 (11–17)	0·31	0·39	0·80

a Definitions of Hayden severity score are as follows: upper respiratory symptom—earache, runny nose, sore throat, and sneezing; lower respiratory symptom—cough, breathing difficulty, hoarseness, and chest pain; systemic symptom—muscle ache, fatigue, headache, and chills; total—all of the above. Symptoms were collected by interview. Severity was ranked on a four‐point scale ranging from none (0) to severe (3). The number of people asked about particular symptoms is reported in parentheses. *P*‐values from Wilcoxon rank‐sum tests comparing distributions of scores by adenovirus species and by age‐group.

**Table 4:** Median severity of reported symptoms among patients with adenovirus‐associated influenza‐like illness over 28‐day study period

	Day 0	Day 3	Day 7	Day 28
Chills	0·5 (42)	0 (16)	0 (20)	0 (27)
Cough	1 (43)	2 (17)	1 (21)	0 (27)
Rhinorrhea	2 (43)	1 (17)	1 (20)	0 (27)
Otalgia	0 (26)	0 (13)	0 (13)	0 (18)
Conjunctivitis	0 (42)	0 (17)	0 (21)	0 (27)
Sneezing	0 (43)	0 (17)	0 (15)	0 (26)
Sore throat	1 (25)	1 (13)	0 (12)	0 (17)
Hoarseness	0 (42)	0 (17)	0 (20)	0 (27)
Dyspnea	0 (40)	0 (17)	0 (20)	0 (27)
Chest pain	0 (25)	0 (13)	0 (12)	0 (16)
Nausea	0 (26)	0 (13)	0 (13)	0 (18)
Vomiting	0 (43)	0 (17)	0 (20)	0 (27)
Diarrhea	0 (42)	0 (17)	0 (20)	0 (26)
Abdominal pain	0 (26)	0 (12)	0 (13)	0 (18)
Loss of appetite	1·5 (42)	1 (17)	0 (19)	0 (27)
Arthralgia	0 (21)	0 (14)	0 (13)	0 (19)
Myalgias	0 (27)	0 (12)	0 (13)	0 (18)
Headache	0 (25)	0 (13)	0 (13)	0 (18)
Fatigue	2 (42)	1 (17)	0 (20)	0 (27)
Vertigo	0 (26)	0 (13)	0 (13)	0 (18)

Symptoms were collected by interview. Severity was ranked on a four‐point scale ranging from none (0) to severe (3). The number of people asked about particular symptoms is reported in parentheses.

Symptom data were recorded for 42%, 47%, and 63% of participants on days 3, 7, and 28, respectively. Analyses of symptom data showed the persistence of some symptoms through day 7, with resolution of virtually all symptoms by day 28 (Table 4). It is worth noting that most patients experienced cough, rhinorrhea, sore throat, loss of appetite, or fatigue at the onset of infection, with rhinorrhea and fatigue being the most severe symptom reported. The median scores for all other symptoms were 0 at enrollment. By day 3, the overall severity of most symptoms declined, with the exception of cough, which increased to moderate severity from mild at baseline. By day 7, the only reported symptoms were cough and rhinorrhea. By day 28, all of the symptoms had resolved.

## Discussion

In a five‐year, observational study of ILI among adults and children, we found that adenovirus‐associated infections were relatively infrequent; only 2·8% of 1536 patients were positive for adenovirus. As expected, the majority of cases were children, as has been previously demonstrated.^13^ Moreover, through the use of a multiplex PCR diagnostic assay, we observed that 50% of cases were positive for at least one other respiratory viral pathogen. Proportions of viral codetection were higher in children, although the frequency of codetections among adults (27%) was notable as well.

The findings of our study also suggest there are differences in prevalence and severity of symptoms by adenovirus species. In general, symptom severity was greater for non‐type C infections. Unfortunately, due to limited numbers of infections, these differences did not reach statistical significance. However, these findings are consistent with those of other studies that non‐C adenovirus infections, specifically serotypes 7 and 14, can be associated with high morbidity and, in some cases, mortality.^14^, ^15^, ^16^ Our B species infections were largely type B3 and generally mild. By contrast, severe cases of type B3 infection in otherwise healthy children have been previously described.^17^ Multiple severe infections with a variety of B subtypes have also been described in military recruits, suggesting that the entire B subgroup may have a higher propensity for severe disease in children and young adults.^12^

Underlying epidemiologic and virologic differences between adenovirus species likely accounts for the findings of our study. While a number of species B adenoviruses have been associated with severe respiratory disease outbreaks among otherwise healthy adults,^5^, ^12^ species C adenoviruses are encountered much earlier in life as common causes of acute respiratory infection among infants and young children.^18^, ^19^ Moreover, species C adenovirus infections are persistent; periods of latent infection, viral reactivation, and prolonged shedding of species C adenoviruses have been described.^20^ This phenomenon of asymptomatic persistent infection may explain the endemic nature of species C adenovirus infection and may also account for observed milder forms of disease due to frequent interaction with the human host.

Overall, the adenovirus‐associated illnesses in our study were mild. There were no deaths, few hospitalizations, and participants reported that symptoms had resolved or substantially declined within 7 days. Therefore, the clinical course of adenovirus‐associated infection described in our study population is similar to those of other upper respiratory viruses.^21^, ^22^

Recruitment and enrollment in our observational study of influenza‐like illness took place in outpatient clinics and inpatient settings of five major military treatment facilities in the United States. Children and spouses of military personnel are eligible to participate and comprised the majority of patients with adenovirus‐associated respiratory infections. As a result, we were not able to accurately assess the burden of adenovirus infection among active duty military personnel, some of whom may have received adenovirus vaccine while in basic training. We did not collect information on participant's prior receipt of adenovirus vaccine. Moreover, the majority of our study sites are not proximal to military training centers where the populations at highest risk for severe respiratory disease due to adenovirus (i.e., military recruits) reside.^6^, ^7^

There are several strengths to this study. First, in an otherwise healthy, majority outpatient population, we described the epidemiology and clinical characteristics of adenovirus‐associated respiratory infections in the context of the overall burden of ILI. The low frequency of solely adenovirus infections, in the absence of underlying medical conditions, suggests the majority of adenovirus infections may be relatively mild in nature, and may not result in patients seeking medical care. Second, our cases stemmed from a multiyear, multisite protocol, thereby minimizing the impact of temporal and/or geographic differences in trends of disease. Third, we utilized a multiplex PCR assay to assess the prevalence of other viral respiratory pathogens. The prevalence of viral codetection was rather high, even among adult patients, and several fold higher than those reported among military recruits.^23^ If these codetections do in fact represent coinfections, it is possible that previous clinical characterizations of adenovirus‐associated illness may have been confounded by the presence of other respiratory viral pathogens.^1^, ^8^, ^24^

An important limitation of this study is the lack of an adenovirus‐positive, asymptomatic comparison group. Because the adenovirus cases were identified from a larger population of patients, all of whom presented with a febrile, influenza‐like illness, we were not able to enroll individuals who were PCR positive for adenovirus yet asymptomatic. Thus, it is possible that some of the observed symptoms were due not to adenovirus, but rather, to coinfecting pathogens, given the propensity of some adenovirus serotypes to cause persistent infection with mild to no clinical illness.

In recent years, multiplex nucleic acid amplification testing has been increasingly used for the etiologic diagnosis of upper and lower respiratory tract infections.^25^, ^26^, ^27^, ^28^, ^29^ These tests have greatly improved the efficiency of clinical microbiology laboratories, as a broad diversity of viral and bacterial pathogens commonly associated with respiratory infections can be detected from a single sample. Although a multiplicity of commercially available assays now exists, comparative evaluations of different platforms have demonstrated a high degree of concordance.^25^, ^29^ Moreover, the use of multiplex assays has shed new light on the relationship between etiology (or etiologies) of illness and clinical severity/outcome; among children with medically attended acute respiratory infection, ~25% were positive for more than one respiratory pathogen,^26^, ^28^ suggesting that complex interactions between multiple viruses, bacteria, and the host may underlie many of the clinical observations associated with these infections.

Our study offers an important perspective on the burden of adenovirus‐associated respiratory illness in the United States military health system. While adenovirus appears to be an infrequent cause of ILI that is relatively mild in nature, our case series investigation demonstrated the propensity for more severe symptoms with non‐type C adenovirus infections. In addition, the observed high prevalence of viral codetection among those with adenovirus‐associated illnesses underscores the importance of using multiplex diagnostic platforms, particularly when elucidating the etiology of respiratory illness and characterizing the severity of symptoms caused by particular pathogens.

## Funding

The study was funded by the National Institute of Allergy and Infectious Diseases, National Institute of Health, under Inter‐Agency Agreement Y1‐AI‐5072, and the Armed Forces Health Surveillance Center, Global Emerging Infections Surveillance and Response System.

## Conflicts of interest

None to report.

## Disclaimer

The views expressed are those of the author(s) and do not necessarily reflect the official views of the Uniformed Services University of the Health Sciences, the United States Navy, the United States Army, the United States Air Force, the United States Department of Defense, nor the Henry M. Jackson Foundation for the Advancement of Military Medicine.

## Appendix 1

The laboratory‐developed molecular diagnostic method is a real‐time PCR assay that targets the penton region of the adenovirus. This assay has been validated using a previously described, CAP‐accredited, conventional PCR assay as the gold standard (12), and is capable of detecting the following serotypes: B3, B7, B14, B21, E4a, E4p, C1, C2, C5, C6, A12, D13, and D30. The validation for the real‐time PCR assay was as follows: Among 100 samples tested (60 adenovirus, 40 influenza), the sensitivity was 100% and the specificity was 90·2%.

The original, conventional PCR assays were CAP‐accredited and validated using viral culture as the gold standard. The first PCR assay was a universal primer set that targeted a 122‐base pair region of the penton gene and had a sensitivity of 100% and specificity of 94·9% when compared to viral culture. The second PCR assay was specific to species B, C, and E and targeted the E1A gene. This assay had a sensitivity of 97·9% and specificity of 94·8% when compared to viral culture. Influenza detection during this time was also done using two laboratory‐developed PCR assays. Both assays were universal primer sets that amplified the 93‐ and 239‐base pair regions of the matrix gene, respectively.
